# Supplementary material for: HBCR_DMR: A Hybrid Method Based on Beta-Binomial Bayesian Hierarchical Model and Combination of Ranking Method to Detect Differential Methylation Regions in Bisulfite Sequencing Data
Source: J Pers Med. 2024 Mar 29;14(4):361. doi: 10.3390/jpm14040361 (PMC11051304; doi:10.3390/jpm14040361)
Supplement: Supplementary file 1 [file jpm-14-00361-s001.zip › S1.pdf]

Table S1. Summary statistics of methylation sequencing data

Sample	Total reads	Mapping Rate	Percentage methylation (CpG context)	Average CpG Methylation	Average CpG Coverage	GC
**T65**	76,723,684	88.50%	47.70%	0.46	24.15	27.04%
**N16**	70,443,130	88.70%	45.70%	0.46	23.53	27.26%
**T20**	67,394,464	88.90%	44.70%	0.44	19.58	27.03%
**N4**	68,165,382	88.80%	46.50%	0.47	22.19	27.19%
**T31**	61,789,306	89.00%	46.90%	0.47	21.69	26.92%
**N10**	57,311,634	89.05%	46.70%	0.47	19.26	27.04%
**T35**	79,004,644	88.90%	46.10%	0.46	24.43	27.11%
**N7**	75,663,274	89.00%	47.20%	0.49	22.62	27.04%
**T45**	64,188,480	89.00%	47.40%	0.47	21.22	27.06%
**N8**	57,091,968	89.80%	46.80%	0.46	20.42	27.41%
**T67**	61,203,576	89.30%	44.30%	0.44	20.77	27.17%
**N14**	66,871,860	89.60%	47.40%	0..47	22.17	27.11%
