# Supplementary material for: HBCR_DMR: A Hybrid Method Based on Beta-Binomial Bayesian Hierarchical Model and Combination of Ranking Method to Detect Differential Methylation Regions in Bisulfite Sequencing Data
Source: J Pers Med. 2024 Mar 29;14(4):361. doi: 10.3390/jpm14040361 (PMC11051304; doi:10.3390/jpm14040361)
Supplement: Supplementary file 1 [file jpm-14-00361-s001.zip › S5.pdf]

Visualization of the highly relevant hypermethylation DMRs with CRC

A. Chr10: [7452243-7452499] Gene symbol: SFMBT2

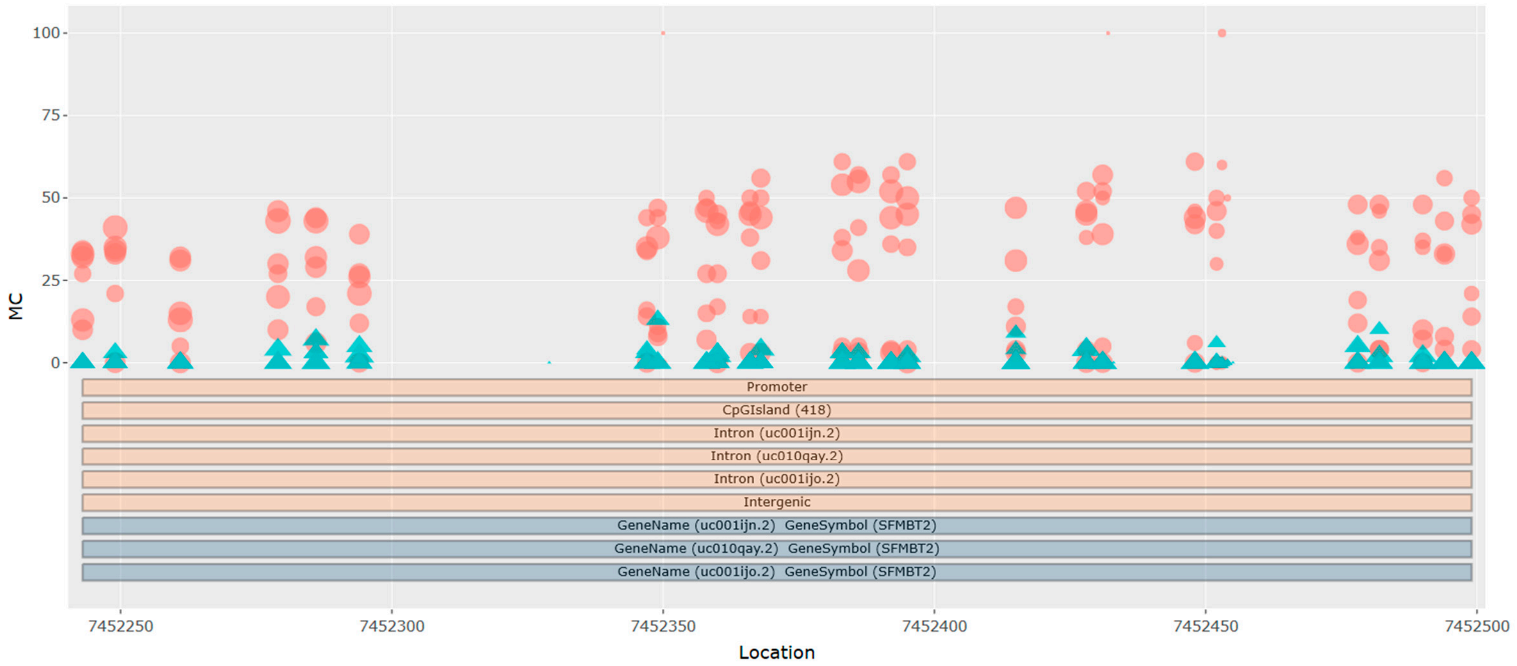

B: Chr12: [24715833-24716098] Gene symbol: SOX5

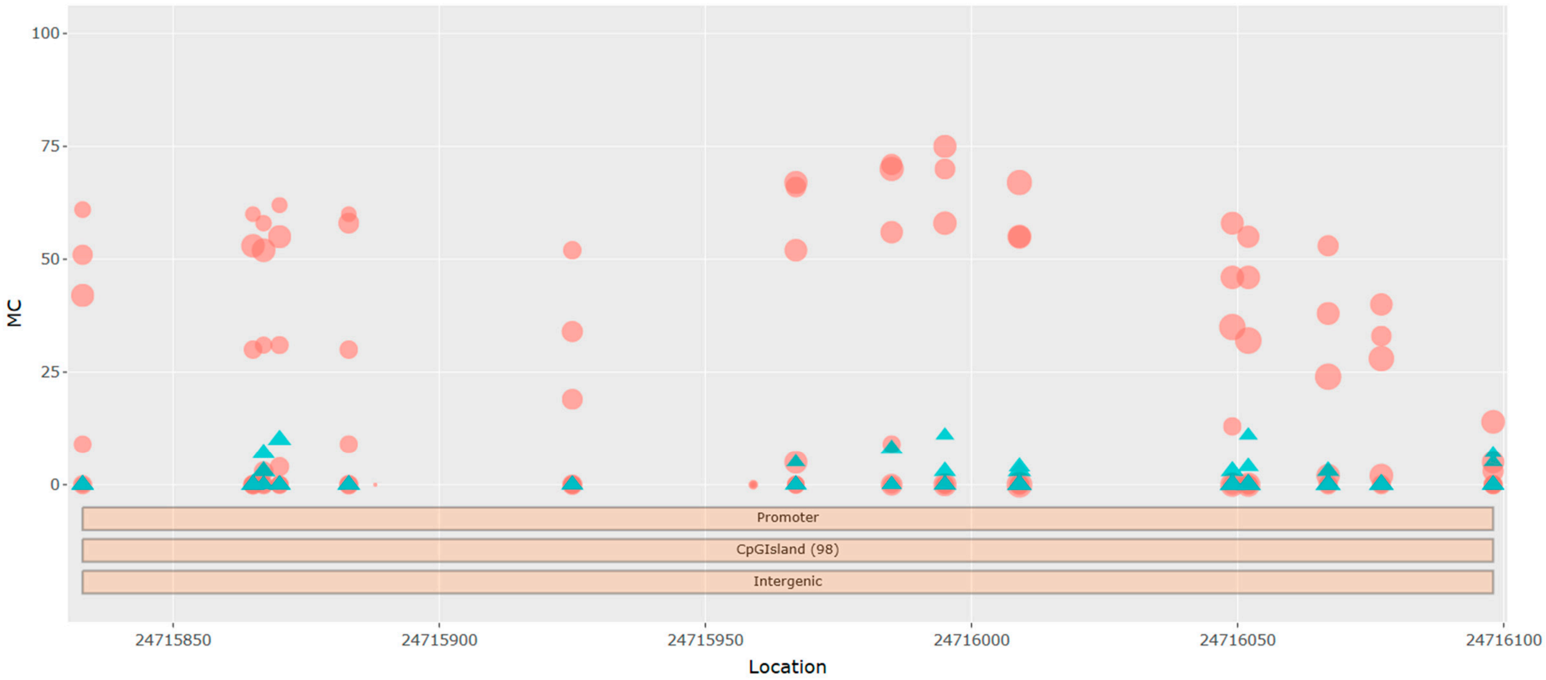

C. Chr19: [22034731-22034990] Gene symbol: ZNF43

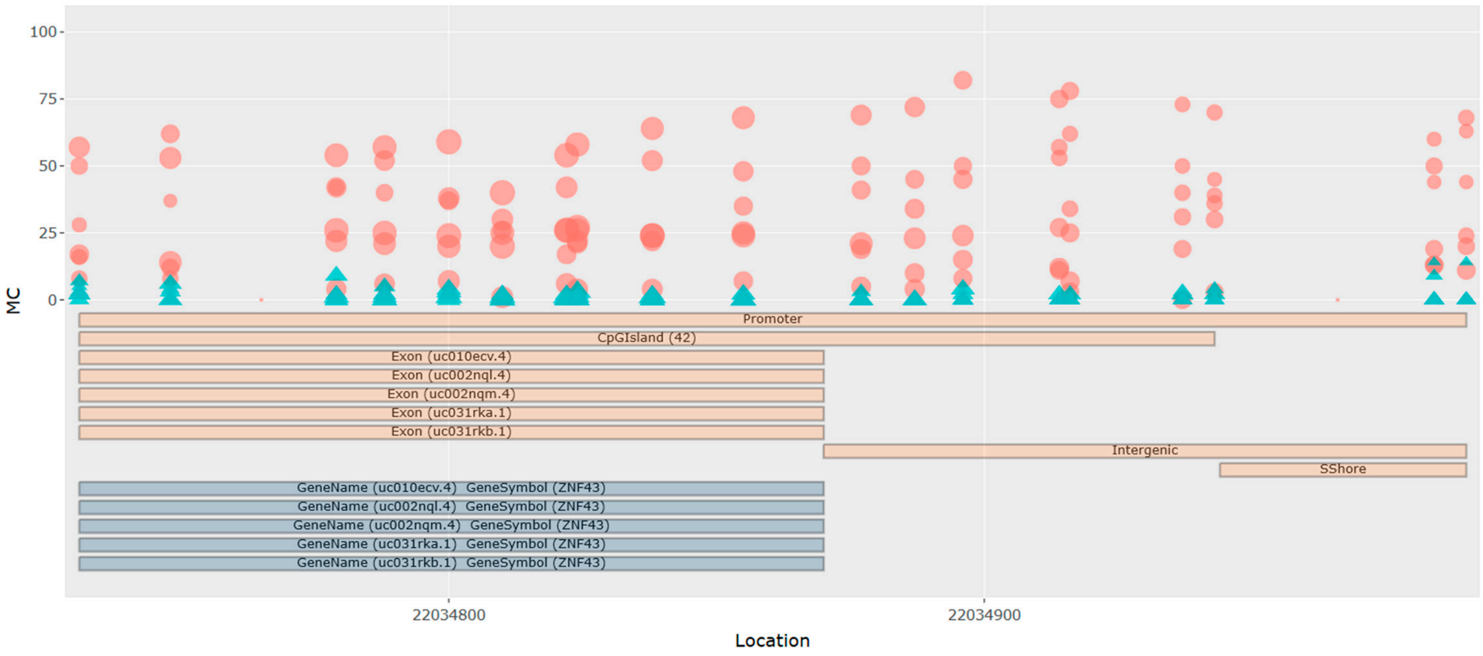

D. Chr1: [49242758-49243000] Gene symbol: AGBL4

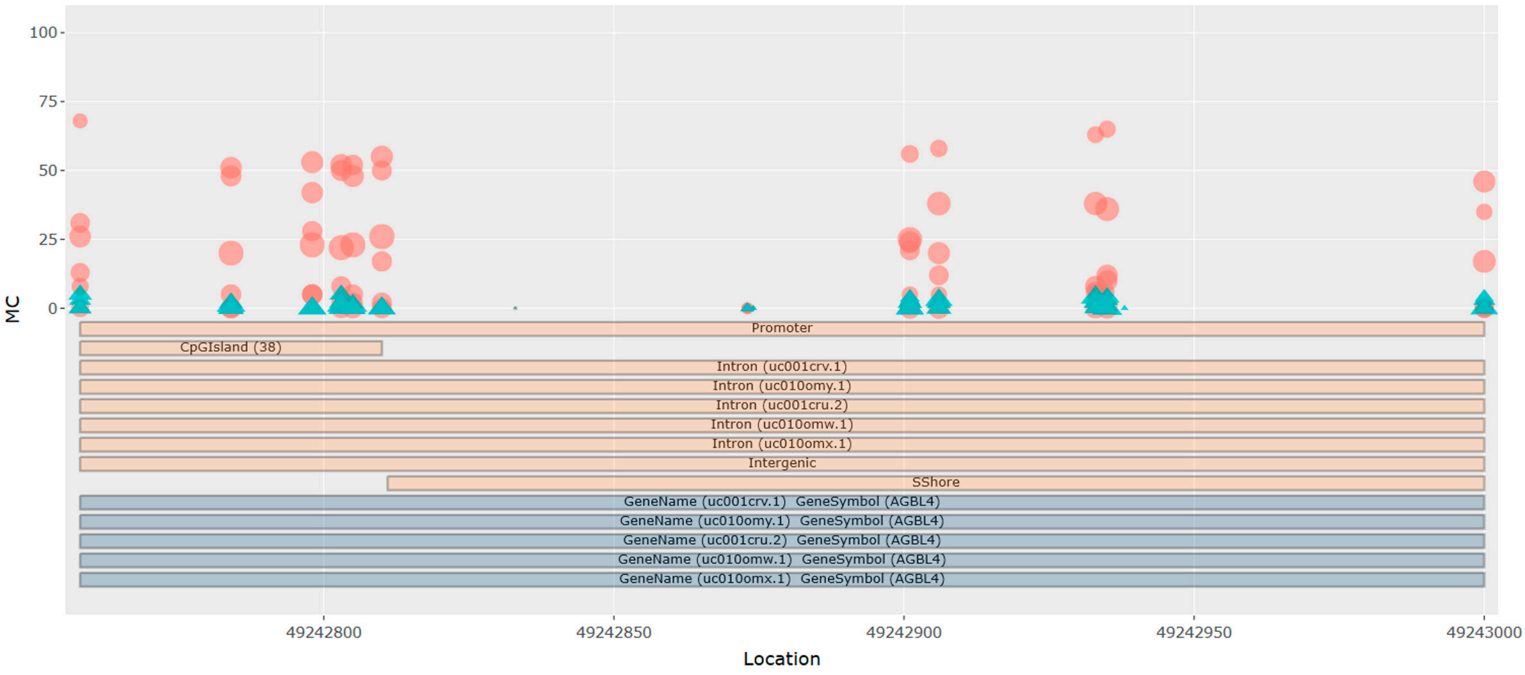

F. Chr12: [24715169-24715370] Gene symbol: SOX5

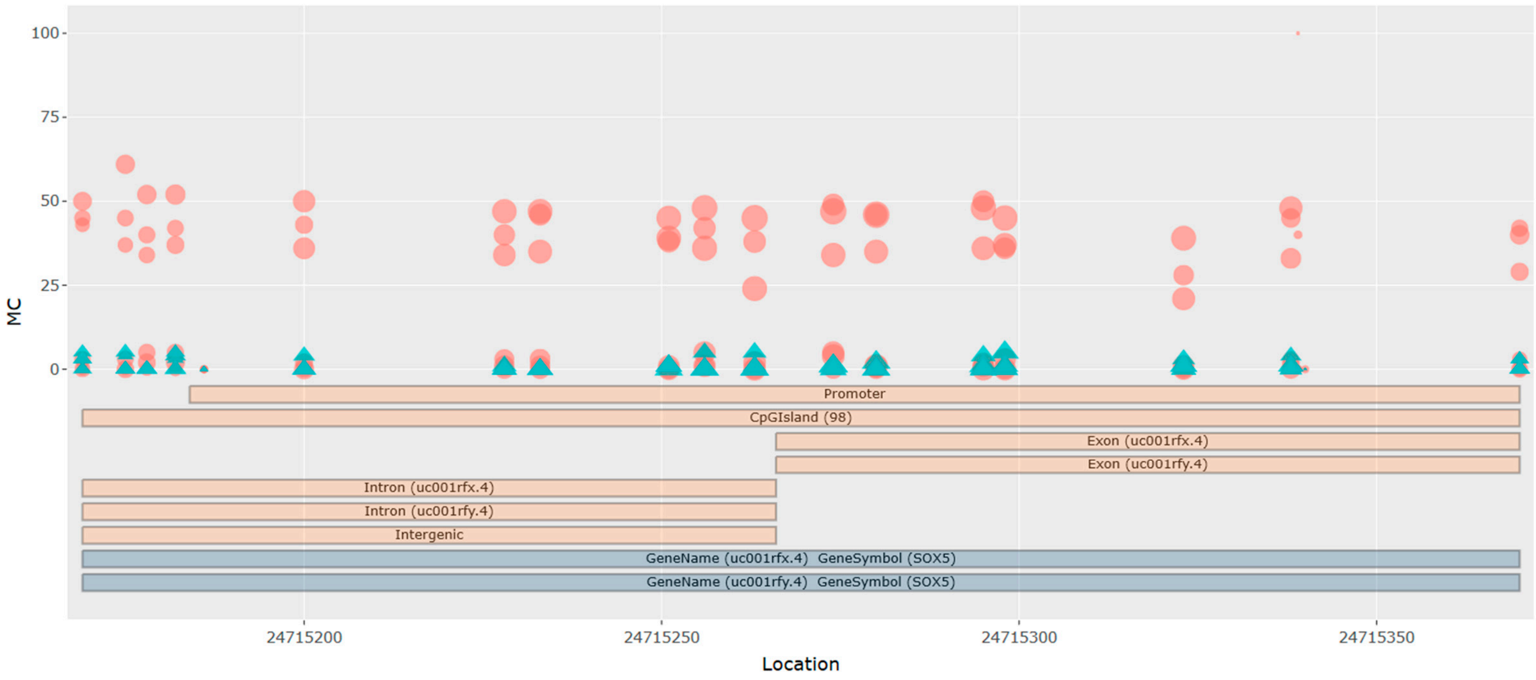

S4: Visualization of the highly relevant DMRs in the top genes A. *SFMBT2*, B. *SOX5*, C. *ZNF43*, D. *AGBL4* and F. *SOX5* in CRC. Pink circles are CRC samples and blue triangulars are control samples
